# New Hospitals and the Medical Profession

**Published:** 1906-03-03

**Authors:** 


### New Hospitals and the Medical Profession.

A proposal to establish a new hospital in any locality is one which demands full and careful discussion. Such a suggestion readily excites a certain degree of social and sentimental enthusiasm, and is an opportunity for the display of local patriotism and pride. But neither of these can provide an adequate defence for an addition to existing hospital organisations, nor will they secure those permanent influences without which a sustained success is impossible. In short, hospital schemes and developments must, in order to obtain justification and the warrant of a favourable issue, rest upon a sound economic basis. They must present in their defence a substantial business claim which can find abundant support in the real necessity of the community. Unless this actually exists, the enthusiasm of the early days will soon prove itself to be a foundation neither strong nor durable. Hence, keen and searching criticism of all new hospital schemes ought to be both welcomed and encouraged.

The statement of this position involves a recognition of the full right of medical practitioners, equally with other citizens, to take part in an inquiry into any suggested new hospital development. Indeed, it is obvious that the local representatives of the medical profession are able on such a topic to speak with peculiar authority, and to contribute to the discussion special and first-hand knowledge. All reasonable persons, therefore, will not only admit, but will welcome the views of the local medical profession when a scheme of hospital development or extension is under the consideration of any community. It must, however, be understood that these views are valuable and are pertinent to the discussion only in so far as they are enlightened by the principles stated in the earlier paragraphs of this article. The medical practitioner has on this question the opportunity to be a specially well-informed witness, and therefore his testimony may be correspondingly valuable. But he is not the arbiter of destiny, and the last word does not rest with him. First, because the hospital question is not, nor can it ever be, solely a medical question. Social, economic, and philanthropic interests and influences have also to be considered. In a word, the question is one, not for any single class, but for the whole community. Secondly, there is danger, or at least there is the possibility, that medical criticism of any new hospital scheme may be determined by the fear that the new proposal will prove prejudicial to the interests of the critics. It is by no means to be said that this consideration is lightly to be put on one side. But what is here urged is this—that whilst this influence is a perfectly proper reason for introducing safeguards and limitations, it is not pertinent to the preliminary discussion whether a hospital shall or shall not be established. That discussion has to deal with two essential principles—the need of the community and the ability, particularly the financial ability, of the district to meet this need. When this has been decided, then is the time for the medical profession to urge that any scheme which may be adopted shall contain provisions to prevent the abuse of the charity of the profession. Until the earlier and essential decision has been taken, medical practitioners have merely the responsibilities and rights of citizens, and if they occupy a special position this is due, not to special interests, but to special knowledge. In so far as they discuss the question on broad and sound principles their views are worthy of peculiar attention. But they make a tactical mistake when they rest their position on the suggestion that their craft is in danger. Some larger and worthier motive is necessary to influence the common judgment and decision. Naturally we have every sympathy with the injury done to the legitimate claims of the medical profession by the abuse of hospital opportunities, as, other questions apart, this means a prostitution of funds intended for the suffering poor; but we believe that the profession in discussing the hospital question is sometimes in danger of taking a too narrow view and of resting its criticisms on an unsound foundation. Hence we define the position as it presents itself to our judgment, and we urge it on the attention of our *confrères* generally, and more particularly of those who, as in certain districts of South London, are now obliged to face the question in the shape of a practical issue.

